# Multiplex immunochromatographic assay using a universal labeling reactant for determining antibiotic residues in milk

**DOI:** 10.14202/vetworld.2024.2527-2536

**Published:** 2024-11-13

**Authors:** Assem N. Jangulova, Nadezhda A. Taranova, Boris B. Dzantiev, Zhannara Zh. Akanova, Aitbay K. Bulashev

**Affiliations:** 1 Department of Veterinary Medicine, Faculty of Veterinary and Livestock Technology, S. Seifullin Kazakh Agrotechnical Research University, 62 Zhenis Avenue, Astana 010011, Kazakhstan; 2 Immunobiochemistry Laboratory, A.N. Bach Institute of Biochemistry, Research Center of Biotechnology, Russian Academy of Sciences, 33, Leninsky Avenue, Moscow 119071, Russia; 3 Department of Microbiology and Biotechnology, Faculty of Veterinary and Livestock Technology, S. Seifullin Kazakh Agrotechnical Research University, 62 Zhenis Avenue, Astana 010011, Kazakhstan

**Keywords:** antibiotic residues, immunochromatographic assay, indirect antibody labeling, limit of detection, milk

## Abstract

**Background and Aim:**

In animal husbandry, antibiotics are frequently used as growth promoters, as well as for illness prevention and treatment. They are considered important toxic and allergenic contaminants of food and a serious risk factor for the spread of antibiotic resistance. National and international regulatory authorities have established limits on the permissible residue of antibiotics in food. Immunochromatographic test strips are the most efficient tools for the simple and rapid control of antibiotics for food safety. In these tests, each antibody specific to a particular antibiotic is labeled with a marker, which complicates the manufacturing technology and increases the cost of the test. This study aimed to develop a multiplex immunochromatographic assay (ICA) to determine streptomycin (STR), tetracycline (TC), and chloramphenicol (CAP) residues in milk by indirect labeling of antibiotic-specific antibodies.

**Materials and Methods:**

Test strips were composed using 15 μm pore size CNPC nitrocellulose membranes, GFB-R4 separation, and AP045 adsorption pads. The applied reactants include TC and STR conjugates with bovine serum albumin, and CAP-soybean trypsin inhibitor conjugate; anti-TC, anti-STR, and anti-CAP mouse monoclonal antibodies; goat anti-mouse immunoglobulin G (GAMI) conjugated with gold nanoparticles (GNPs) and staphylococcal protein A. Milk samples were collected from cows and goats that had not been injected with any antibiotics. STR and TC/CAP at concentrations of 0.27–600 ng/mL and 0.04–30 ng/mL were added to skim milk, respectively. Milk samples were tested by ICA and calibration curves were constructed to determine the sensitivity of the assay for each antibiotic used.

**Results:**

A multiplex ICA of three antibiotic residues in milk was achieved through labeling of immune complexes using a single reagent, GNPs-GAMI. The visual limits of detection (LOD) were 600 ng/mL, 10 ng/mL, and 30 ng/mL for STR, TC, and CAP in cow and goat milk, respectively. Instrumental LODs gave higher sensitivity when analyzed goat milk to STR, TC, and CAP (1.2, 0.05, and 1.3 ng/mL) than cows’ milk (7.27, 0.96, and 2.07 ng/mL, respectively).

**Conclusion:**

The developed approach for manufacturing multiplex ICA tests for the detection of antibiotic residues in milk does not involve labeling specific antibodies and is implemented using only GNP conjugates with anti-species antibodies.

## Introduction

In livestock production, antibiotics are frequently used to cure and prevent diseases in animals and to enhance development and productivity. Antibiotics and other antimicrobial medications are mostly used in dairy production to treat mastitis and other infectious diseases [1, 2]. Antibiotic usage is predicted to rise globally by 67% [3] to over 100,000 tons between 2010 and 2030, mostly because intense large-scale animal agriculture requires the use of antibiotics [4]. Antibiotics are vital for maintaining animal welfare and cannot be replaced in veterinary medicine soon due to the lack of suitable alternatives [5]. Antibiotic residues are transmitted into the human body through animal products, posing health risks [6, 7]. Anaphylaxis and allergic responses are common ways in which antibiotic side effects manifest themselves. In addition, they can lead to an imbalance in the bacterial flora of the intestine and induce cancer, mutagenesis, and teratogenesis [8, 9]. In addition, one of the biggest risks to public health related to the unchecked use of antibiotics is the development of drug resistance [10], which is a major issue [11]. For this reason, the World Health Organization, as well as other food safety authorities (European Food Safety Organization, the US Food and Drug Administration, Codex Alimentarius, etc.) have established maximum residue levels (MRLs) for antibiotics in products of animal origin [12, 13]. Exceeding the MRL level poses not only a potential risk to consumer health but also creates great problems for producers of fermented milk products because it inhibits the activity of starter cultures [14].

Control of residual antibiotic amounts is especially important in developing countries, including the Republic of Kazakhstan, where more than 70% of milk comes for processing from private farms and farmsteads [15], where the holding time for cows after antibiotic injection is not always respected. Consequently, the protection of consumer health requires rapid and sensitive methods for determining the safety of milk in terms of antibiotic residue [16]. The most common analytical techniques for identifying antibiotics in food include microbiological analysis, high-performance liquid chromatography, liquid chromatography coupled with mass spectrometry, and immunoassays. The microbiological method is relatively not labor-intensive, but the results can be obtained no earlier than 3–4 h [17]. Chromatographic techniques provide information on antibiotic residues with high accuracy [18], and they are used only in laboratories with expensive equipment and trained personnel [19, 20]. For screening food products for contamination by antibacterial drugs, enzyme-linked immunosorbent assay (ELISA) is becoming increasingly common as a relatively fast and inexpensive method. This test is recommended by European Union Directive 2002/657 for the determination of residues in veterinary drugs in animal products [21]. However, ELISA requires several incubation and washing steps, and the assay usually requires more than 1 h [22]. In addition, the ELISA kit cannot simultaneously detect two or more analytes [23]. Therefore, practical applications require simpler-to-use but sufficiently sensitive rapid tests that allow simultaneous determination of several antibiotic residues. To date, a number of multiplex immunochromatographic assay (ICA)-based tests have been proposed for this purpose [24, 25], which use a mixture of several conjugates of antibiotic-specific antibodies with a marker [26–28]. Over the past decade, a number of studies have reported on the use of ICA to detect antibiotic residues in milk [29–31], as well as in other foods [17, 19]. In our previous study by Jangulova *et al*. [32], multiplex ICA was proposed to simultaneously detect three antibiotics. However, this approach has several limitations: high consumption of specific reagents, decrease in antibody specificity, insufficient sensitivity of the analysis, and complications of the technology for manufacturing the test system. These problems can be overcome by the indirect introduction of a colorimetric marker, for example, through anti-species antibodies [33]. Urusov *et al*. [34] suggested that indirect labeling of mycotoxins-specific antibodies overcomes the limitations of the ICA and makes it possible to increase sensitivity up to 20 times compared with the traditional principle. Similar results were reported by Hendrickson *et al*. [35]. In previous studies, ampicillin and tetracycline [33], aflatoxin B1 and T-2 toxin [34], and tylosin and lincomycin [35], with known concentrations in buffer but not in milk, were used as analytes. Moreover, the developed test strips detected these contaminants separately [33, 34] and/or simultaneously in a double ICA [35].

This study aimed to develop a multiplex ICA of streptomycin (STR), tetracycline (TC), and chloramphenicol (CAP) residues in raw cow and goat milk with indirect labeling of specific antibodies.

## Materials and Methods

### Ethical approval

This study was approved by the Ethics Committee of S. Seifullin Kazakh Agrotechnical Research University (Protocol No 2, November 1, 2023). Milk collection and animal welfare were performed under the supervision of a veterinarian.

### Study period and location

The study was conducted from November 2023 to June 2024 at the Faculty of Veterinary and Livestock Technology, S. Seifullin Kazakh Agrotechnical Research University and at the Research Center of Biotechnology, A.N. Bach Institute of Biochemistry.

### Materials and instrumentation

TC, STR, and CAP bases were purchased from Sigma-Aldrich (St. Louis, MO, USA). Mouse anti-TC monoclonal antibodies (mAbs) and TC conjugates with bovine serum albumin (BSA) were obtained from Eximio Biotec (Wuxi, China). The All-Russian Research Center for Molecular Diagnostics and Treatment provided the anti-CAP and anti-STR mAbs. The conjugation of CAP with a soybean trypsin inhibitor (STI) and STR with BSA was performed using the technique outlined by Byzova *et al*. [36]. The source of goat anti-mouse immunoglobulin G (GAMI) was Arista Biologicals, Pennsylvania, USA. The supplier of sucrose was Cristalco in Paris, France. Sigma-Aldrich provided the BSA, tetrachloroauric acid (HAuCl4), tannic acid, sodium citrate, Tween-20, Triton X-100, Tris, protein A, and sodium azide. We bought NaCl, K_2_PO_4_, KOH, and other chemicals (such as solvents, acids, and salts) from Chimmed in Moscow, Russia. Distilled water was purified by the Sartorius Arium^®^ pro system in Göttingen, Germany; all solutions for syntheses and tests were prepared with distilled water. The manufacture of test strips was carried out using nitrocellulose membrane CNPC 15 MDI Easypack kits, separation membrane GFB-R4, and absorption membrane AP045 (Advanced Microdevices, Ambala Cantonment, India).

### Preparation of gold nanoparticles (GNPs)

Synthetic methodology, as described by Byzova *et al*. [37], was used for the preparation of GNPs. Deionized water (97.5 mL) was mixed with 1 mL of 1% HAuCl_4_ (Sigma-Aldrich, St. Louis, USA). After bringing the reaction liquid to a boil, 1.5 mL of a 1% sodium citrate solution (Sigma-Aldrich) was added while stirring. After 25 min of boiling, the mixture was cooled and kept at 4–6°C until needed. Using spectrophotometry (Biochrom, Cambridge, UK) at 400–800 nm and transmission electron microscopy (TEM) (JEOL, Tokyo, Japan), the size and form of the produced GNPs were evaluated.

### Synthesis of antibody conjugates with GNPs

Before conjugating anti-species antibodies and GNPs, sodium carbonate was used to reduce the latter’s pH to 8.5. Conjugation was completed in accordance with the methodology outlined by Hayat [38]. To the GNP solution, mAbs (10 μg/1 mL of GNPs) were added dropwise after dilution in 10 mM Tris buffer (pH 8.5) (Sigma-Aldrich). The mixture was constantly stirred for 30 min. Next, BSA was added to a final concentration of 0.25% and incubated for an additional 15 min at room temperature (20ºC). Centrifugation was used to precipitate the resultant mixture for 15 min at 13,400× *g*. After suspending the pellet in Tris buffer containing BSA and sucrose, sodium azide was added to a final concentration of 0.05%.

### Preparation of immunochromatographic test strips

The adsorption pad AP045, a functional nitrocellulose CNPC membrane with a pore size of 15 microns, and a plastic backing are all included in the MDI Easypack kit (Advanced Microdevices, Ambala Cantonment, India). Using an Iso Flow automatic dispenser (Imagene Technology, Lebanon, USA), agents were rendered immobile on the membranes at a rate of 0.12 μL/mm. Conjugates of TC-BSA, STR-BSA, and CAP-STI created the test zone, whereas protein A formed the control zone. The reagent concentrations were as follows: 1 mg/mL of TC-BSA conjugate, 0.25 mg/mL of STR-BSA, 0.3 mg/mL of CAP-STI, and 1 mg/mL of Protein A in phosphate-buffered saline (PBS). A PBS solution containing 1% sucrose and 0.25% BSA was used as the sorption medium. At least 20 h were spent drying the membrane with the applied chemicals at 20ºC. Conjugate and sample membranes were not included in the test strip design. Following the assembly of the ICA components, the membrane sheet was divided into 3.0-mm-wide test strips using an Index Cutter-1 (A-Point Technologies, USA), and it was then kept in a sealed package with silica gel until it was needed between 20°C and 22 °C [39].

### Milk sample preparation

Milk samples were collected from Holsteinized black-and-white cows and Saanen goats that had not been injected with antibiotics. Known antibiotic amounts were added to skim milk through centrifugation at 5000× *g*/4 min. After mixing for 5 min in a rotary mixer, 100 μL samples were collected for testing [40]. The test milk samples contained STR and TC/CAP at concentrations of 0.27–600 ng/mL and 0.04–30 ng/mL, respectively.

### Immunochromatography

The ICA procedure was performed at 20ºC. Test strips were submerged in solutions that included (i) antibiotics at various concentrations, (ii) GNP conjugates with GAMI (GNPs-GAMI) in a dilution corresponding to optical density (OD) OD522 = 1.0, and (iii) mAbs against STR (0.15 μg/mL), TC (1.0 μg/mL), and CAP (0.2 μg/mL). The results were recorded after 10 min. For statistical processing, all measurements were performed in triplicate.

The specificity of the homemade ICA test was tested on 90 milk samples from cows kept on a dairy farm free of infectious diseases and not treated with antibiotics or other antibacterial drugs in comparison with a commercial lateral flow test (Pioneer Meizheng Bio-tech, Beijing, China).

### Statistical analysis

Immunochromatographic results were processed as described by Taranova *et al*. [41]. Strips were scanned using an Epson Perfection V600 Photo scanner (Epson, Suwa, Japan) at 600 dpi without contrast or color correction modes. The color intensity of the test and control lines was calculated using TotalLAB software (Nonlinear Dynamics, Newcastle, UK). The dependence of color intensities in relative units on antibiotics concentration (C) was determined using TotalLAB software (Cleaver Scientific, Rugby, UK). The instrumental limit of detection (LOD) was determined as the analyte concentration at which the test zone staining intensity exceeded according to the following formula [42]: LOD = X 3S, where X is the average color intensity of the blank and S is the standard deviation of the blank.

### Recovery determination

Recovery for milk samples spiked with antibiotics was determined as a percentage using the following formula [43]: R = A*100/N, where R is the degree of recovery, %; A is the detected content of the analyte; and N is the content of the added analyte.

## Results

### Preparation and characterization of GNPs

**Figure-1:**
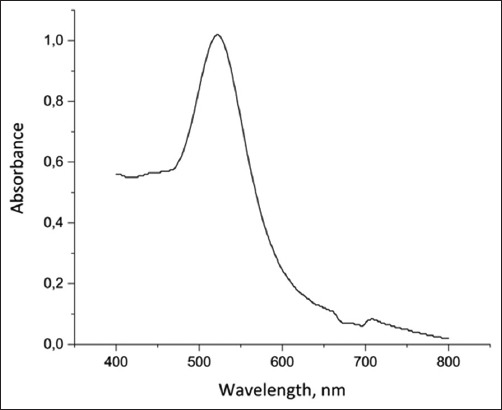
Absorption spectra of the gold nanoparticles.

To achieve high ICA sensitivity, the characteristics of the markers used to label the antibody and/or antigen play an important role. In this study, we used spherical GNP particles as a marker because they are chemically inert, are characterized by stability, high sorption capacity, and ease of synthesis of homogeneous particles of the same size. The GNP precursors were characterized by spectrophotometric and microscopic methods. When studying the absorption of the nanoparticles at 400–800 nm, a peak was detected at 520 nm (Figure-1).

**Figure-2:**
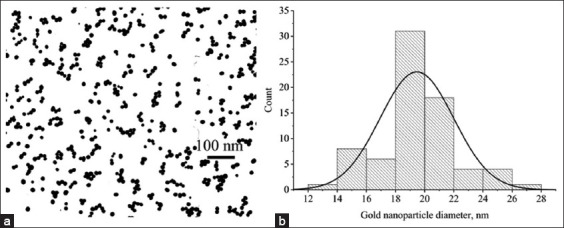
(a) Fragment of a TEM micrograph of GNPs and (b) a histogram of the GNP sample size distribution. GNPs=Gold nanoparticles, TEM=Transmission electron microscopy (JEOL, Tokyo, Japan).

The TEM results showed that the prepared GNPs are homogeneous, not aggregated, and their shape is close to spherical (Figure-2a), and a histogram of the size distribution was plotted (Figure-2b).

The average size of GNPs in the sample was 19.47 ± 2.53 nm (minimum value – 13.96 nm, maximum value – 26.65 nm) with an elongation factor of 1.21 ± 0.19.

### Immunochromatographic test design

**Figure-3:**
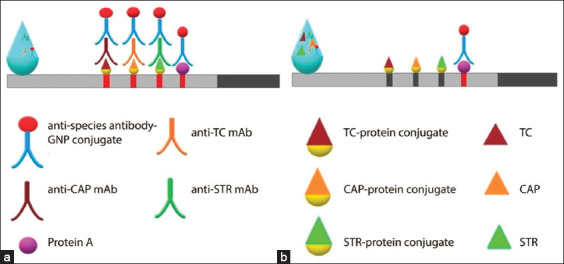
The principle of competitive ICA for the detection of antibiotic residues in milk using unlabeled specific antibodies ([a] The sample does not contain antibiotic residues; [b] The sample contains STR, CAP, and TC). ICA=Immunochromatographic assay, STR=Streptomycin, TC=Tetracycline, CAP=Chloramphenicol.

To increase the sensitivity of the analysis and maintain the intensity of staining in the detection lines [44], we used indirect conjugation of the marker with antibiotic-specific mAbs using GNPs-GAMI, that is, specific antibodies were used in free (unlabeled) forms. The principle of the ICA test is illustrated in Figure-3.

The end of the test strip opposite the absorption pad was dipped into a milk sample containing mAbs against STR, TC, and/or CAP, and GNP-GAMI as the secondary antibodies. If antibiotics are present in the sample, they will bind to the specific antibodies, thereby hindering their interaction with the immobilized antibiotics in the test lines and preventing the subsequent formation of a colored band by labeled secondary antibodies. If antibiotics are not present in the sample, the specific antibodies will bind to the test lines and will be displayed by GNP-GAMI in the form of a colored band/s on the membrane.

The sensitivity of ICA largely depends on the selection of the optimal ratio of reactants. We used various concentrations of mAbs (600, 300, 150, 75, 37.5, 18.7, 9.4, and 4.7 ng/mL) and different ODs of GNP-GAMI (4, 8, and 13.3 units) in a 100-μL analyte volume.

The anti-species antibody concentration was varied from 5 to 20 µg/1 mL of GNPs. The optimal concentration was 10 µg/1 mL of GNPs. The selected antibody concentration allowed the formation of immunoglobulin polylayers on the nanoparticle surface, resulting in the formation of the maximum analytical signal and minimum background signal. The probe volume was varied from 50 µL to 200 µL. The optimal milk volume was 100 µL, ensuring complete antigen detection and optimal analysis time.

**Figure-4:**
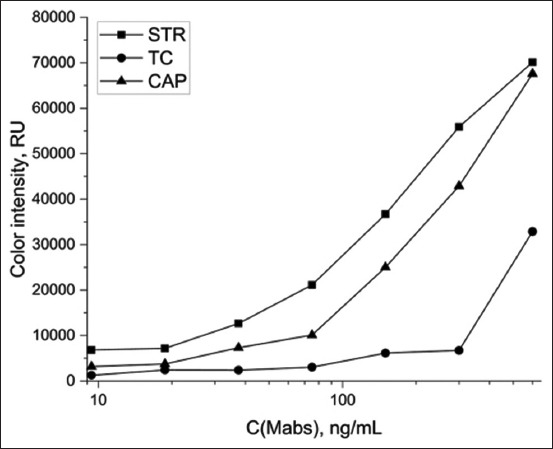
Dependence of color intensity of test line on concentration of specific monoclonal antibodies detected by the GNP-GAMI (arrows indicate the selected parameters). GNP-GAMI=Gold nanoparticles-Goat anti-mouse immunoglobulin G.

Figure-4 shows the color intensity of the test lines as a function of the concentration of STR-, TC-, and CAP-specific mAbs.

The selection of the optimal concentration of specific antibodies is based on the formation of an analytical signal that is 10% higher than a blank optical signal. As can be seen, the best detection of STR, CAP, and TC by mAbs indirectly labeled with GNP-GAMI (OD = 8 units) was achieved at antibody concentrations of 75, 100, and 500 ng/mL, respectively.

**Figure-5:**
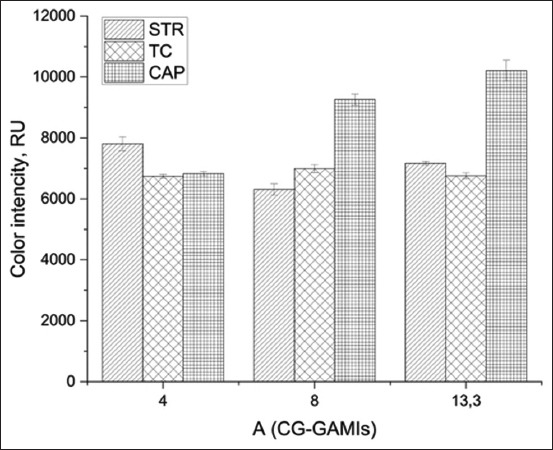
Dependence of the analytical signal for STR, CAP, and TC on GNPs-GAMI conjugate’s OD (arrows indicate the selected parameters). STR=Streptomycin, TC=Tetracycline, CAP=Chloramphenicol, GNP-GAMI=Gold nanoparticles-goat anti-mouse immunoglobulin G, OD=Optical density.

The optimal OD of GNP-GAMI was determined by adding the conjugate with an OD of 4, 8, or 13.3 units to milk containing the above concentrations of mAbs (Figure-5).

Histograms show that the color intensity of the test lines differed markedly at different OD values for the conjugate. Thus, at an OD of 8 units, the color intensity of the CAP test line was lower than that at 13.3 units but higher than that of the TC and STR. Therefore, the optimal OD can be considered to be 8 units when testing milk for three antibiotics at the same time.

### Determination of the analytical characteristics of the ICA test

**Figure-6:**
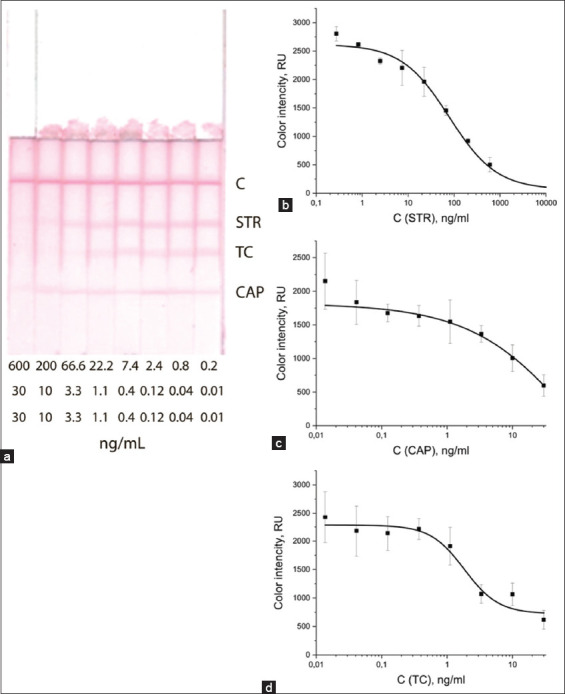
Images of test strips after determining antibiotic residues in cow (a) milk and calibration curves for (b) STR, (c) CAP, and (d) TC in competitive ICA. ICA=Immunochromatographic assay, STR=Streptomycin, TC=Tetracycline, CAP=Chloramphenicol.

**Figure-7:**
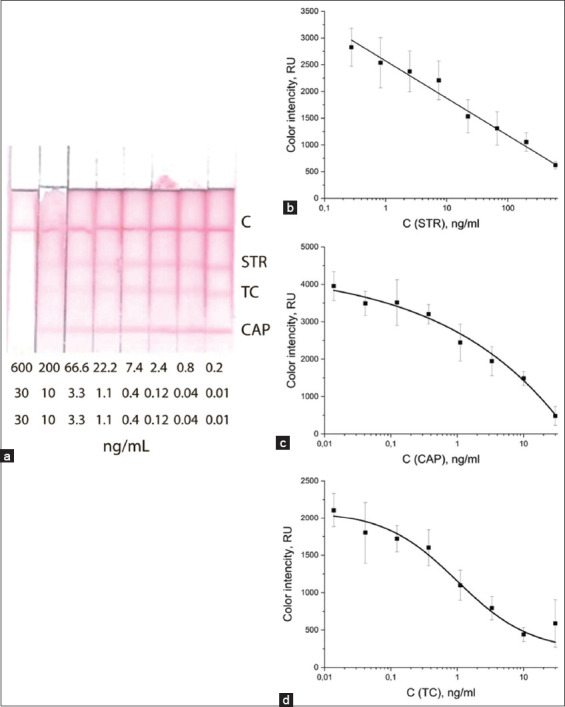
Images of test strips after determining the three antibiotic residues in goat (a) milk and calibration curves for (b) STR, (c) CAP, and (d) TC in competitive ICA. ICA=Immunochromatographic assay, STR=Streptomycin, TC=Tetracycline, CAP=Chloramphenicol.

After choosing the optimal conditions, test strips prepared for the determination of STR, TC, and CAP were tested in triplicate on cow’s and goat’s milk samples. Figures-6 and 7 show antibiotic calibration curves in competitive ICA and the appearance of test strips after analysis of cow’s and goat’s milk samples containing analytes at various concentrations: STR from 600 ng/mL to 0 ng/mL, and TC and CAP from 30 ng/mL up to 0 ng/mL.

The analytical characteristics of the developed test were calculated based on the graphs shown in Figures-6 and 7 and are presented in Table-1.

**Table-1:** Analytical characteristics of the ICA.

Antibiotics	Instrumental LOD (ng/mL)		Visual LOD (ng/mL)	
	
	Cow milk	Goat milk	Cow milk	Goat milk
Streptomycin	7.27	1.2	600	600
Tetracycline	0.96	0.05	10	10
Chloramphenicol	2.07	1.3	30	30

ICA=Immunochromatographic assay, LOD=Limits of detection

The visual LOD values in the milk of both species of animals were the same; however, the instrumental LOD gave higher sensitivity to the test when analyzing goats’ milk.

Examination of milk from healthy cows (n = 90) not injected with antibiotics and/or other drugs showed negative results for the presence of antibiotic residues in both prepared and commercial ICA tests.

A criterion for the diagnostic value of ICA tests is the recovery (R) degree of the analyte from the food matrix. This indicator was determined using samples of cows’ milk with the addition of a certain number of antibiotics (Table-2).

**Table-2:** Recoveries of antibiotic residues in spiked cow milk samples using ICA and a commercial ELISA kit (Gold Standard Diagnostics, Budapest, Hungary).

Addition amount (ng/mL)	Detected amount ± SD (ng/mL)		Recovery ± SD (%)	
	
	ICA	ELISA	ICA	ELISA-kit
STR				
200	195.0 ± 4.0	197.0 ± 1.0	97.0 ± 2.0	98.0 ± 0.5
66.7	69.0 ± 2.0	67.0 ± 1.0	103.0 ± 3.0	100.0 ± 1.0
22.2	23.0 ± 1.0	23.0 ± 2.0	105.0 ± 5.0	105.0 ± 9.0
TC				
30	28.0 ± 2.0	29.0 ± 2.0	94.0 ± 9.0	98.0 ± 5.0
3.3	3.1 ± 0.2	3.4 ± 0.2	95.0 ± 5.0	103.0 ± 4.0
1.1	1.2 ± 0.1	1.0 ± 0.1	103.0 ± 5.0	94.0 ± 5.0
CAP				
30	29.0 ± 2.0	> 0.2*	96.0 ± 6.0	NA
10	9.7 ± 0.1	> 0.2	97.0 ± 1.0	NA
3.3	3.2 ± 0.1	> 0.2	97.0 ± 3.0	NA

* =ELISA kit does not determine the amount of CAP if it exceeds 0.2 ng/mL; NA=Not applicable, ICA=Immunochromatographic assay, STR=Streptomycin, TC=Tetracycline, CAP=Chloramphenicol, ELISA=Enzyme-linked immunosorbent assay, SD=Standard deviation

Table-2 presents the assay recoveries for milk samples spiked with antibiotics. The developed ICA test was not inferior for this parameter to a commercial ELISA kit, showing high degrees of analyte recoveries (94%–105%).

## Discussion

Antibiotics are now widely used to ensure animal welfare and increase the efficiency of livestock farming. Antimicrobial agents are added to feed premixes to prevent diseases and stimulate animal growth. They are also used in the form of injectable medications. Therefore, providing a growing population of the planet with safe livestock products has become the most important task of veterinary science and practice. Tetracyclines and aminoglycosides, including STR, are among the antibiotics widely used to treat mastitis and other infectious diseases in cows. The use of CAP, the most toxic of the antibiotics, is banned in the European Union and the Eurasian Economic Union, although unscrupulous suppliers may use it not only for treatment, but also to destroy pathogenic flora in milk, as well as to increase its shelf life by adding it directly to the product [45]. Reliable food antibiotic control drives the increasing demand for simple, rapid, and inexpensive tests for detecting drug-derived contaminants. ICA, sometimes called lateral flow assay or ICA, is the basis for these assays, which use colloidal gold-labeled antibiotic-specific antibodies, and some of them are available on the veterinary market for the control of beta-lactams, TC, STR, and CAP in milk (e.g., Delvotest BLF, Proqui-test 4, and Betastar 4D). However, these tests are still not used for food safety laboratories in developing countries due to their high cost. Therefore, we need express diagnostic tools that would be competitive in the market for veterinary drugs, not only in terms of sensitivity and specificity but also in terms of price.

One possible way to reduce costs is to simplify the manufacturing of ICA tests. This problem, in our opinion, can be solved by indirect labeling of immunoglobulins specific to antibiotics using secondary antibodies coupled to the marker. The assay’s sensitivity was shown to be 80 times higher when intact neomycin-specific immunoglobulins were used, and secondary-labeled antibodies were used to detect the antibody-antibiotic combination in ICA [46]. GNP-labeled secondary antibodies were successfully used to improve the sensitivity of ICA to aflatoxin B1 [47], aflatoxin B1 and T-2 toxin [34]. These variants exhibited significantly lower detection limits than traditional ICA using the same reagents. As the authors note, an important advantage of the new ICA variant is the reduction in the consumption of specific antibodies, which are much more expensive than secondary antibodies. Moreover, marker-labeled anti-species secondary antibodies are versatile reagents that can be used for the detection of various analytes.

Unlike the above methods, in our study, we developed a multiplex ICA for monitoring milk safety for three antibiotic residues (STR, TC, and CAP) simultaneously. Second, our test strip does not contain a pad for GNP-labeled anti-species antibody, which was used in the architecture of neomycin detection [46], and does not involve two sequential incubations of test strips (first in the test sample with specific antibodies, and then in a solution of secondary antibody conjugated with GNPs), as in the case of ICA for aflatoxin B1 [47]. Multiplex ICA was performed in a single step by immersing the test strip in an analyte containing both specific antibodies against STR, TC, and CAP, as well as secondary-labeled anti-species antibodies, and allowing results to be obtained within 10 min.

The ICA methods allow the detection of STR [48], TC, oxytetracycline, chlortetracycline [49], and oxytetracycline [40] in milk in the range of 15.0–30.0 ng/mL. The developed multiplex ICA has an instrumental LOD of 7.3 and 0.9 ng/mL for STR and TC in cows’ milk, and in goat’s milk, these antibiotics are detected at concentrations of at least 1.2 and 0.1 ng/mL, respectively. Thus, our test is not inferior in sensitivity to the described analogs in terms of the specified developments and the range of practical needs. The detection limits achieved correspond to the established standards [50]. Moreover, the indirect introduction of the marker allows the use of a universal immunoreagent, thereby reducing the amount of specific antibodies and increasing the concentration of the colored marker in the analytical zone.

Due to the simultaneous incubation of anti- biotic-specific antibodies with GNPs-GAMI in milk samples, the single-stage nature of the developed ICA makes it more attractive than known methods. However, the use of antibodies in solution form requires appropriate storage to maintain their specificity and affinity, which poses a constraint in veterinary practice. In this regard, further study aimed at constructing an ICA format in which all reactants are collected on the test strip is of interest for “point-of-care” diagnostics.

## Conclusion

A rapid ICA was developed for detecting STR, TC, and CAP based on indirect labeling of anti- biotic-specific antibodies with GNPs. The results encourage further study to improve ICA to make it more available for food safety monitoring at milk collection points and food markets. Instrumental LOD made it possible to detect lower antibiotic amounts than visual ones and to determine the quantitative content of each analyte in a milk sample. The developed multiplex ICA had instrumental LODs of 7.3, 0.9, and 2.1 ng/mL in cows’ milk and 1.2, 0.1, and 1.3 ng/mL in goats’ milk for STR, TC, and CAP, respectively. In addition, using GNP-labeled anti-species antibodies, the proposed test technique can lower the consumption of specific antibodies. The latter are suitable for testing milk for different antibiotic residues and are universal for mono- or polyclonal antibodies.

## Authors’ Contributions

BBD: Conceptualization and supervision. ANJ, NAT, and ZZA: Methodology, validation, and formal analysis. ANJ and NAT: Investigation and drafted the manuscript. AKB: Interpreted the results and revised the manuscript. All authors have read and approved the final version of the manuscript.
